# Digital Biopsy and Network Analysis of Dynamic [^68^Ga]Ga-FAPI-46 Data in Patients with Malignant and Benign Pancreatic Lesions

**DOI:** 10.2967/jnumed.125.270185

**Published:** 2026-02

**Authors:** Manuel Röhrich, Frederik M. Glatting, Magdalena Geisinger, Anna-Maria Spektor, Hans-Georg Buchholz, Joel Wessendorf, Isabelle von Goetze, Jorge Hoppner, Jakob Liermann, Maximilian Knoll, Matthias Lang, Ulrike Heger, Mathias Schreckenberger, Martin Loos, Adriana Tavares, Klaus Herfarth, Jürgen Debus, Uwe Haberkorn, Mark G. Macaskill

**Affiliations:** 1 Department of Nuclear Medicine, University Hospital Mainz, Mainz, Germany;; 2 Department of Radiation Oncology, University Medical Center Mannheim, Medical Faculty Mannheim, University of Heidelberg, Mannheim, Germany;; 3 DKFZ-Hector Cancer Institute, University Medical Center Mannheim, Mannheim, Germany;; 4 Department of Nuclear Medicine, University Hospital Heidelberg, Heidelberg, Germany;; 5 Department of Radiation Oncology, University Hospital Heidelberg, Heidelberg, Germany;; 6 Department of Surgery, University Hospital Heidelberg, Heidelberg, Germany;; 7 Centre for Cardiovascular Science, University of Edinburgh, Edinburgh, United Kingdom;; 8 Edinburgh Imaging, University of Edinburgh, Edinburgh, United Kingdom;; 9 German Center of Lung Research, Heidelberg, Germany; and; 10 Clinical Cooperation Unit Nuclear Medicine, German Cancer Research Center, Heidelberg, Germany

**Keywords:** [^68^Ga]Ga-FAPI, pancreatic ductal adenocarcinoma, clustering, dynamic PET

## Abstract

The pathologies pancreatic ductal adenocarcinomas, inflammatory lesions of the pancreas, postpancreatectomy reactive tissue, and recurrent pancreatic ductal adenocarcinomas all express fibroblast activation protein and are hardly distinguishable by static PET using [^68^Ga]Ga-labeled fibroblast activation protein inhibitors (FAPIs) combined with CT. Dynamic imaging allows full [^68^Ga]-Ga-FAPI kinetic profile analysis, highlighting differences among these pathologies. Here, we applied a voxel-level digital biopsy approach combined with network analysis and clustering to characterize healthy, nonmalignant pathologic, and malignant pathologic kinetic signatures. **Methods:** This monocentric, retrospective study included 47 patients (>18 y) with morphologically unclear pancreatic lesions on CT or MRI and supplemental [^68^Ga]Ga-FAPI-46 PET/CT in a primary (31 patients) or recurrent (16 patients) setting. Lesions were classified according to biopsy results (primary cases) or CT appearance and clinical course (recurrent cases). Digital biopsy samples (300 voxels) of pancreatic lesions and control organs (muscle, fat, kidneys, liver, and blood) were taken and then masked and imported into an open source visual analytics application. Voxel networks were created with multiple digital biopsy samples from a single scan or digital biopsy samples combined from multiple scans, with a minimum Pearson correlation value of 0.7. A *k*–nearest-neighbor edge reduction was applied before Markov clustering. Datasets were then unmasked for interpretation. Static PET parameters (SUV_max_ and SUV_mean_) and time to peak of pancreatic lesions and control tissues were extracted from isotropic volumes and analyzed by a *t* test (threshold for significance, *P* = 0.05). **Results:** This work created 47 individual networks and 2 combined networks. Within individual networks, voxels tended to arrange and cluster within the sampled volume of interest (VOI; left and right kidneys strongly coclustered). Networks typically arranged into healthy controls, elimination organs, and pathologic (malignant and nonmalignant) regions. Pathologies tended to cluster with high purity (>95% from the same VOI), with multiple clusters per VOI, indicating intralesional heterogeneity. Our analysis approach could differentiate between malignant and nonmalignant pathologies in the primary and recurrence settings. This differentiation was driven by slower FAPI clearance within malignant voxels. **Conclusion:** The kinetics of [^68^Ga]Ga-FAPI-46 across the different tissues, coupled with this sampling and analysis approach, allowed the separation and identification of healthy, nonmalignant pathologic, and malignant pathologic clusters and kinetic features that may facilitate diagnosis and warrant further investigation.

## Visual Abstract

**Figure:**
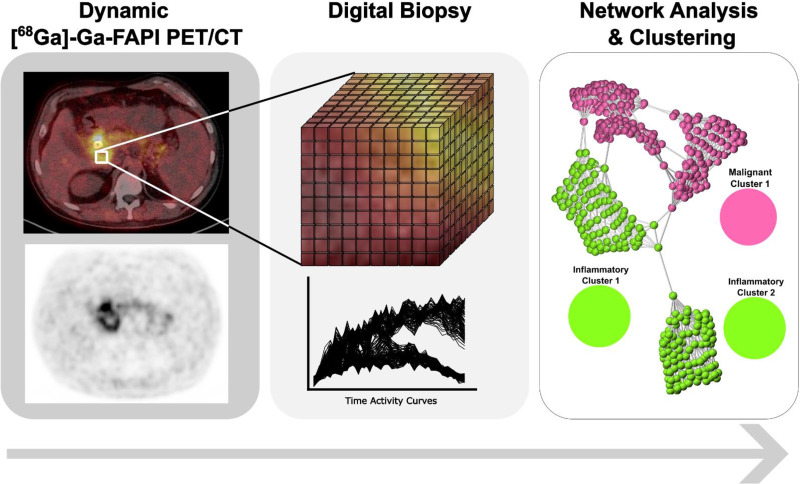

Numerous recent studies have demonstrated the high clinical potential of PET using [^68^Ga]Ga-labeled fibroblast activation protein inhibitors (FAPIs) combined with CT for tumor staging and therapy planning in general (1) and for pancreatic ductal adenocarcinomas (PDACs) in particular (2–4). However, distinguishing pathologies such as PDAC, inflammatory lesions of the pancreas (ILPs), postpancreatectomy reactive tissue (PRT), and recurrent PDAC (RPDAC) remains a diagnostic challenge, because all of these lesions show variably intense FAPI uptake, which can be caused by cancer-associated fibroblasts of a primary PDAC or RPDAC (4,5), activated fibroblasts within inflammatory processes of the pancreas, or reactive changes after pancreatic surgery (4,6,7). In addition, nonspecific pancreatic FAPI uptake has been described (8). Recent publications from our group have described the differential kinetic behavior of fibroblast activation protein tracers within malignancies and inflammatory or reactive changes of the pancreas (9,10) and other tissues (11). We have demonstrated that differences in tracer uptake over time from multiple time points and dynamic PET imaging may reflect the presence of these different fibroblast activation protein–avid subtypes of fibroblasts in malignant and inflammatory or reactive pathologies (4,12). Analysis of tracer uptake over time can highlight the differences among these pathologies, in addition to static imaging results (4,9,12). The optimal information on tracer uptake over time can be achieved through dynamic PET scanning, resulting in time–activity curves that visualize signal intensities of selected volumes of interest (VOIs) with high temporal resolution. However, analysis of dynamic PET data is challenging and difficult to integrate into clinical practice, because the interpretation of time–activity curves, which usually features qualitative classification (tumor-typical or not), requires the individual reader to have significant experience and is affected by subjectivity.

The utility of network analysis for the assessment of dynamic PET has already been demonstrated in the preclinical setting (13). That study assessed ^18^F-FDG bone metabolism and demonstrated that data-driven network analysis of dynamic PET scans is suitable for the description of complex networks defined by their time–activity curve. In our study, we evaluate the use of a voxel-level digital biopsy approach combined with network analysis and clustering in a dataset of a cohort of oncologic patients with suspected primary PDAC or RPDAC. This approach has the potential to mitigate the limitations caused by reader dependency in the interpretation of dynamic FAPI PET data. We also present a pipeline for this data-driven, image interpretation approach. We hypothesize that this approach will allow the identification of healthy, nonmalignant pathologic, and malignant pathologic kinetic signatures, which could facilitate diagnosis and clinical decision making in terms of improved differentiation of PDAC and its important differential diagnoses.

## MATERIALS AND METHODS

### Patients

This monocentric retrospective study includes 47 patients with unclear pancreatic lesions, defined by the absence of CT or MRI morphologic signs highly suggestive for malignancy (double duct sign and vessel enhancement) or benign lesions (diffuse calcification and duct-penetrating sign) (14,15). For the study, 31 patients were examined in a primary setting and 16 patients were examined in a recurrence setting after pancreatic surgery. All patients underwent additional dynamic [^68^Ga]Ga-FAPI-46 PET/CT imaging. All patients were individually referred to additional [^68^Ga]Ga-FAPI-46 PET for characterization of pancreatic lesions or for tumor staging by their treating physicians because of inconclusive findings in CT or MRI. Written informed consent was obtained individually from all patients, following the regulations of the German Pharmaceuticals Act §13(2b). Retrospective analysis of PET data and clinical and pathologic data was approved by the local institutional review board (study number S-115/2020). [^68^Ga]Ga-FAPI PET imaging data of this dataset have been partially included in previous publications of our group (10,16,17).

### Dynamic [^68^Ga]Ga-FAPI PET/CT Scans

Synthesis and labeling of [^68^Ga]Ga-FAPI-46 were conducted as described in previous publications (18–20). PET scans were performed using a Biograph mCT Flow scanner (Siemens Healthineers), according to published protocols (21). Dynamic PET data of the upper abdomen were acquired and consisted of 28 time frames that started directly after injection of [^68^Ga]Ga-FAPI-46 (2 MBq/kg) and continued until 60 min after injection. The frame duration increased progressively from 30 s to 10 min during the scanning time. Images of the PET dynamic series were reconstructed iteratively using the ordered-subsets expectation maximization 3-dimensional algorithm (matrix, 400 × 400) with 5 iterations and 21 subsets, including point-spread function and time of flight. For all patients, dynamic and static PET scans were acquired directly after tracer injection and 60 min after injection of [^68^Ga]Ga-FAPI-46, respectively.

### Lesion Classification

For patients in the primary setting, pancreatic lesions were histologically confirmed by surgical resection or biopsy after [^68^Ga]Ga-FAPI-46 PET imaging in 24 of 31 cases. After surgery, patients were screened for possible recurrence using CT, physical examinations, and laboratory values (carbohydrate antigen 19-9, C-reactive protein, lipase, and amylase) for at least 18 mo. Lesions of these patients were classified as recurrences, ILPs, or reactive changes according to previously published criteria (10): In CT, space-occupying, progredient lesions with narrowing of surrounding vessels were considered typical for local recurrences and masses in areas of surgery without growth, compression, or infiltration of surrounding structures were considered typical for reactive tissue. None of the cases in the recurrence setting were confirmed by histology.

### Data Homogenization and Digital Biopsy

To achieve homogeneous PET/CT data, the voxel size of all PET images was transformed to 1 × 1 × 4 mm. To minimize motion artifacts, image-based motion correction was applied in each case. For standardized digital biopsy, cube-shaped VOIs consisting of 300 voxels were chosen to provide a sufficient number of voxels per sample, as well as a reasonable sample volume for anatomically correct data extraction from small-volume pathologies or healthy tissues. FAPI-positive lesions were defined by a threshold of a tumor-to-background ratio (compared with the aortic blood pool) of 1.5 in the last frame of the dynamic dataset. FAPI-positive pancreatic lesions, healthy-appearing kidneys and liver (as excretion organs and controls), and blood, fat, and muscle (as control organs) were sampled based on CT morphology by a board-certified nuclear medicine physician, a board-certified radiologist, and a doctorate student. To analyze signal intensities, SUV_max_ and SUV_mean_ were extracted from isotropic volumes within static PET images (40–60 min). For data homogenization, biopsy delineation and data extraction PMOD software version 4.4 (PMOD Technologies LLC) was used.

### Network Analysis

Voxelwise dynamic data of pancreas pathologies and healthy tissues were extracted using PMOD version 4.4 and masked before importing into Graphia version 3.1 (Supplemental Fig. 1 [supplemental materials are available at http://jnm.snmjournals.org]) (22). Voxel networks were created for individual patient analysis, with voxels from multiple VOIs of pathologic lesions and healthy tissues of a single scan of each patient, and for lesion-based analysis, with voxels from VOIs of pancreatic lesions combined from multiple patients, with a minimum Pearson correlation value of 0.7. A *k*–nearest-neighbor edge reduction, and a minimum component size of 10 for individual networks or 100 for combined networks, was applied before Markov clustering. The parameters (minimum Pearson value, *k*–nearest neighbor, and cluster granularity) in the resulting networks were adjusted to provide the best separation between nodes while incorporating as many nodes as possible within the main structural network. The range of parameters for networks consisting of data from a single scan was 0.7–0.78 for the minimum Pearson value, 7–12 for *k*–nearest neighbor (descending rank order), and 1.2 for cluster granularity. The parameters for both networks consisting of data from multiple scans was 0.95 for the minimum Pearson value, 25 for *k*–nearest neighbor (descending rank order), and 1.3 for cluster granularity. The parameters for each network are shown in Supplemental Table 1. After optimization of the networks, the datasets were unmasked for interpretation.

### Statistical Analysis

Static and dynamic parameters of pancreatic lesions (PDAC vs. ILP and RPDAC vs. PRT) were analyzed by Mann–Whitney *U* test, with a threshold for significance of *P* equal to 0.05 using GraphPad Prism version 10.0 (GraphPad Software). Biodistribution of pancreatic lesions and physiologic tissues was displayed as bar charts, and parametric comparisons of types of lesions were displayed by box plots with medians, interquartile ranges, and single values.

## RESULTS

### Results of Lesion Classifications

**FIGURE 1.**
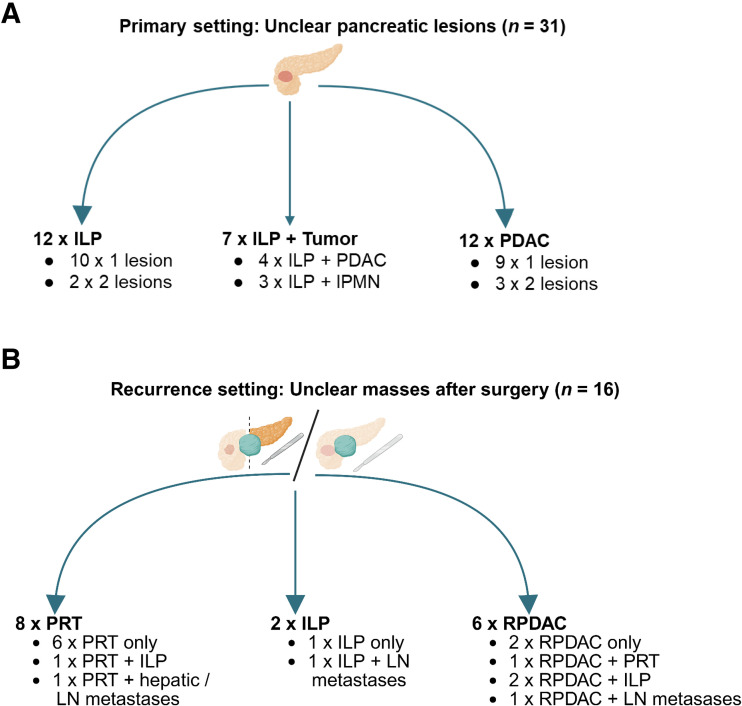
Distribution of lesion classifications in primary (A) and recurrence (B) settings. IPMN = intraductal papillary mucinous neoplasm of pancreas; LN = lymph node. (Created using BioRender.)

In the primary setting, 12 patients had only ILP (1 or 2 lesions), 7 had ILP with a PDAC or intraductal papillary mucinous neoplasms, and 12 patients had a PDAC (1 or 2 lesions). In the recurrence setting, reactive tissue was present in 8 patients (in 1 with ILP and in 1 with hepatic and lymph node metastases), ILP was found in 2 patients (in 1 with lymph node metastases), and 6 patients had an RPDAC, as depicted in Figure 1. Table 1 provides a patientwise overview of the clinical data, classifications, and classification methods for all 68 lesions in 47 patients (age, 32–80 y; average age, 62 y).

**TABLE 1.** Patient Characteristics

Patient	Sex	Age (y)	Clinical scenario	Lesion no.	Lesion localization	Lesion classification	Classification method
1	M	44	Primary	1	Pancreatic head	ILP	Histology
				2	Pancreatic body	ILP	Histology
2	F	67	Primary	1	Pancreatic body	PDAC	Histology
				2	Pancreatic body	PDAC	Histology
3	M	60	Primary	1	Pancreatic head	ILP	Histology
				2	Pancreatic body	IPMN	Histology
4	M	41	Primary	1	Pancreatic body	ILP	Histology
5	M	57	Primary	1	Pancreatic head	ILP	Histology
6	M	37	Primary	1	Pancreatic tail	ILP	Histology
				2	Pancreatic head	PDAC	Histology
7	M	61	Primary	1	Pancreatic head	ILP	Histology
8	M	67	Primary	1	Pancreatic head	PDAC	Histology
				2	Pancreatic head	PDAC	Histology
				3	Pancreatic lymph node	LN METs	Histology
9	M	77	Primary	1	Pancreatic tail	ILP	CT/clinical course
				2	Pancreatic head	IPMN	Histology
10	M	66	Primary	1	Pancreatic head	ILP	CT/clinical course
				2	Pancreatic body	ILP	CT/clinical course
11	M	73	Primary	1	Pancreatic tail	ILP	CT/clinical course
				2	Pancreatic head	IPMN	Histology
12	M	53	Primary	1	Pancreatic head	ILP	Histology
13	F	72	Primary	1	Pancreatic head	ILP	CT/clinical course
14	M	72	Primary	1	Pancreatic tail	ILP	CT/clinical course
				2	Pancreatic head	PDAC	Histology
15	M	61	Primary	1	Pancreatic head	PDAC	Histology
				2	Pancreatic body	PDAC	Histology
16	M	37	Primary	1	Pancreatic head	PDAC	CT/clinical course
17	M	71	Primary	1	Pancreatic head	ILP	Histology
18	M	32	Primary	1	Pancreatic tail	ILP	Histology
19	M	75	Primary	1	Pancreatic tail/body	PDAC	Histology
20	M	77	Primary	1	Pancreatic tail	PDAC	Histology
21	M	69	Primary	1	Pancreatic tail	PDAC	Histology
22	F	73	Primary	1	Pancreatic head/body	PDAC	Histology
23	M	43	Primary	1	Pancreatic head	ILP	Histology
24	M	56	Primary	1	Pancreatic tail	PDAC	Histology
25	F	59	Primary	1	Pancreatic head	ILP	Histology
				2	Pancreatic tail	PDAC	Histology
26	M	51	Primary	1	Pancreatic tail	ILP	Histology
27	M	55	Primary	1	Pancreatic head	ILP	Histology
28	M	72	Primary	1	Pancreatic body	PDAC	Histology
29	F	69	Primary	1	Pancreatic head	PDAC	Histology
30	M	68	Primary	1	Pancreatic tail	PDAC	Histology
31	M	64	Primary	1	Pancreatic tail	ILP	Histology
				2	Pancreatic head	PDAC	Histology
32	M	50	Recurrence	1	Pancreatic head	Reactive	CT/clinical course
33	F	62	Recurrence	1	Pancreatic head	Reactive	CT/clinical course
34	M	66	Recurrence	1	Pancreatic head	ILP	CT/clinical course
34	M	66	Recurrence	2	Parasplenic lymph node	LN METs	CT/clinical course
35	M	80	Recurrence	1	Pancreatic head	RPDAC	CT/clinical course
36	F	69	Recurrence	1	Pancreatic body	Reactive	CT/clinical course
				2	Pancreatic head	RPDAC	CT/clinical course
37	M	53	Recurrence	1	Pancreatic tail	Reactive	CT/clinical course
38	F	72	Recurrence	1	Pancreatic tail	ILP	CT/clinical course
39	M	61	Recurrence	1	Pancreatic tail	ILP	CT/clinical course
				2	Pancreatic head	RPDAC	CT/clinical course
40	F	64	Recurrence	1	Pancreatic tail	ILP	CT/clinical course
				2	Pancreatic head	RPDAC	CT/clinical course
41	M	66	Recurrence	1	Pancreatic body	Reactive	CT/clinical course
42	M	73	Recurrence	1	Pancreatic head	Reactive	CT/clinical course
43	M	59	Recurrence	1	Pancreatic head	ILP	CT/clinical course
				2	Pancreatic head	Reactive	CT/clinical course
44	M	49	Recurrence	1	Hepatic segment IV	HEP METs	Histology
				2	Prehepatic lymph node	LN METs	Histology
				3	Pancreatic head	Reactive	CT/clinical course
45	M	72	Recurrence	1	Paraaortic lymph node	LN METs	Histology
				2	Pancreatic head	RPDAC	CT/clinical course
46	M	59	Recurrence	1	Pancreatic head	RPDAC	CT/clinical course
47	M	70	Recurrence	1	Pancreatic head	Reactive	CT/clinical course

IPMN = intraductal papillary mucinous neoplasm; LN METs = lymph node metastases; HEP METs = hepatic metastases.

### Static Signal Intensities

**FIGURE 2.**
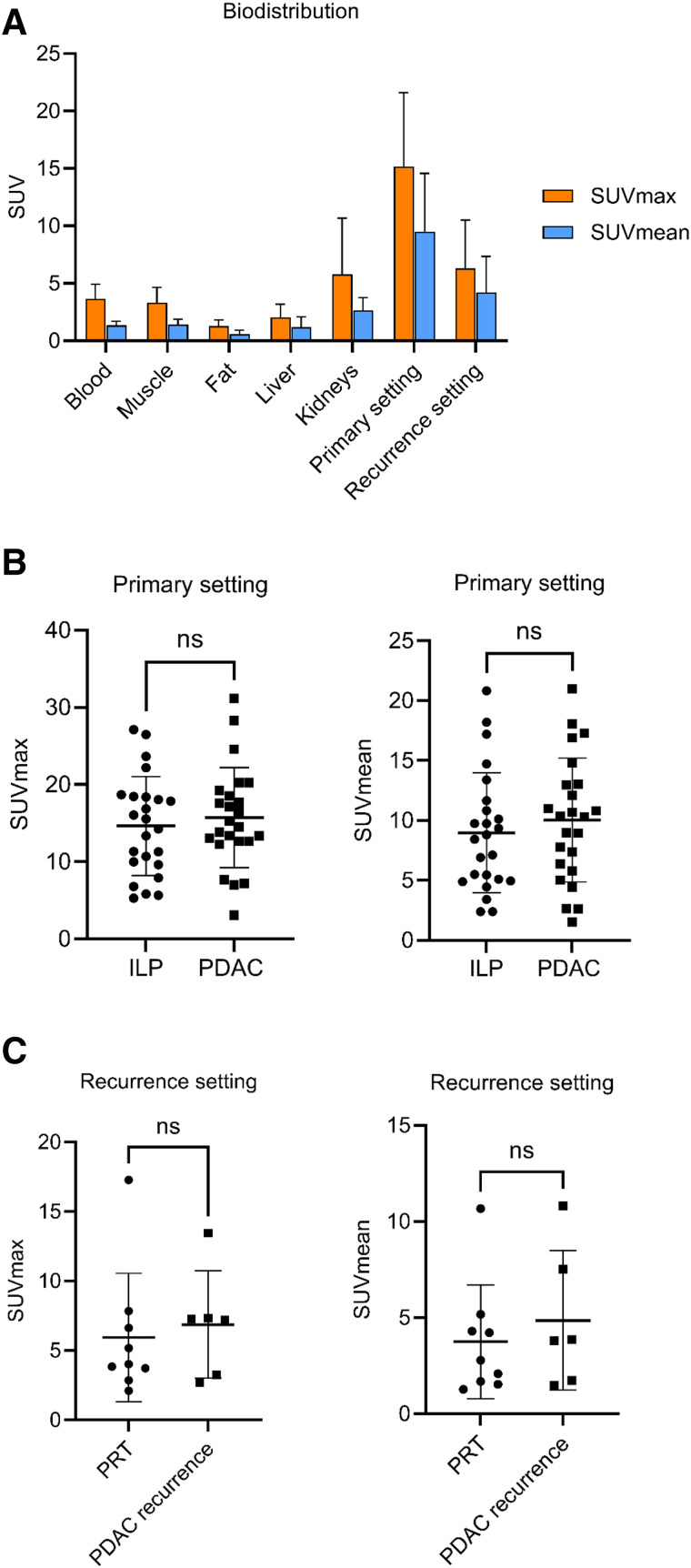
Static imaging data of primary PDAC, RPDAC, ILP, and PRT using [^68^Ga]Ga-FAPI-46. (A) Biodistribution of healthy tissues and pancreas lesions displayed as average SUV_max_ and SUV_mean_ (±SD). (B) SUV_max_ and SUV_mean_ of PDAC and ILP in primary setting (31 scans). (C) SUV_max_ and SUV_mean_ of RPDAC and PRT (16 scans). Dots and squares show individual SUV_max_ and SUV_mean_, horizontal lines show averages, and whiskers display SD (B and C). ns = not significant.

The static signal intensities (40–60 min after injection) of all pancreatic lesions were markedly elevated compared with healthy tissues except kidneys (Fig. 2A). Primary pancreatic lesions showed distinctively higher FAPI uptake than pancreatic lesions in the recurrence setting, which accounts for primary PDAC compared with RPDAC (average SUV_max_, 15.71 vs. 6.87; *P* = 0.0037l; average SUV_mean_, 10.03 vs. 4.862; *P* = 0.03), as well as for ILP compared with PRT (average SUV_max_, 14.64 vs. 5.94; *P* = 0.0008; average SUV_mean_, 8.978 vs. 3.75; *P* = 0.006; Figs. 2B and 2C). According to pronounced overlap of signal intensities, no significant differences between SUV_max_ and SUV_mean_ of PDAC compared with ILP (Fig. 2B) and RPDAC compared with PRT were demonstrated (Fig. 2C).

### Time–Activity Curve Characteristics of PDAC and Benign Differential Diagnoses

**FIGURE 3.**
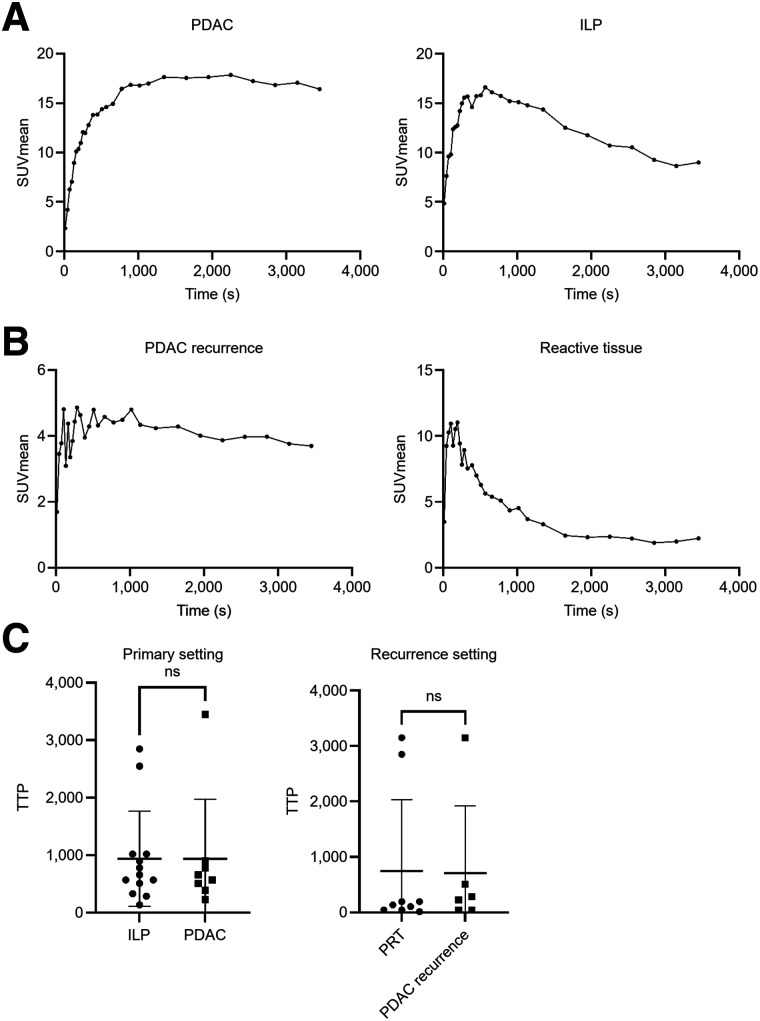
Dynamic imaging data of primary PDAC, RPDAC, ILP, and PRT using [^68^Ga]Ga-FAPI-46. (A) Exemplary time–activity curves in primary setting showing uptake of [^68^Ga]Ga-FAPI-46 over time in PDAC and ILP. (B) Exemplary time–activity curves in recurrence setting showing uptake of [^68^Ga]Ga-FAPI-46 over time in RPDAC and reactive tissue. (C) Comparison of average time to peak (±SD) between ILP and PDAC in primary setting (31 scans) and PRT and RPDAC in recurrence setting (16 scans). Dots and squares show individual time-to-peak values, horizontal lines show averages, and whiskers display SD. ns = not significant; TTP = time to peak.

Visual analysis of the time–activity curves of primary PDAC, RPDAC, ILP, and reactive changes after an operation revealed qualitative curve characteristics, which were typically enriched in each differential diagnosis (Figs. 3A and 3B): either primary PDAC or RPDAC showed prolonged tracer accumulation with a relatively late peak of activity and only moderate washout over time. In contrast, ILP showed an early peak followed by rapid and continuous washout. For reactive changes, early time to peak followed by marked washout, even more pronounced than in ILP, was observed in most cases. Prototypical time–activity curves of primary PDAC, ILP, RPDAC, and reactive tissue are shown in Figures 3A and 3B. Although the exemplary time–activity curves showed different behavior over time for PDAC compared with ILP in the primary setting and RPDAC compared with PRT in the recurrence setting, the conventional time-to-peak time–activity curve parameter did not show significant differences between malignant and benign pathologies in the primary or in the recurrence setting (Fig. 3C).

### Individual Networks

**FIGURE 4.**
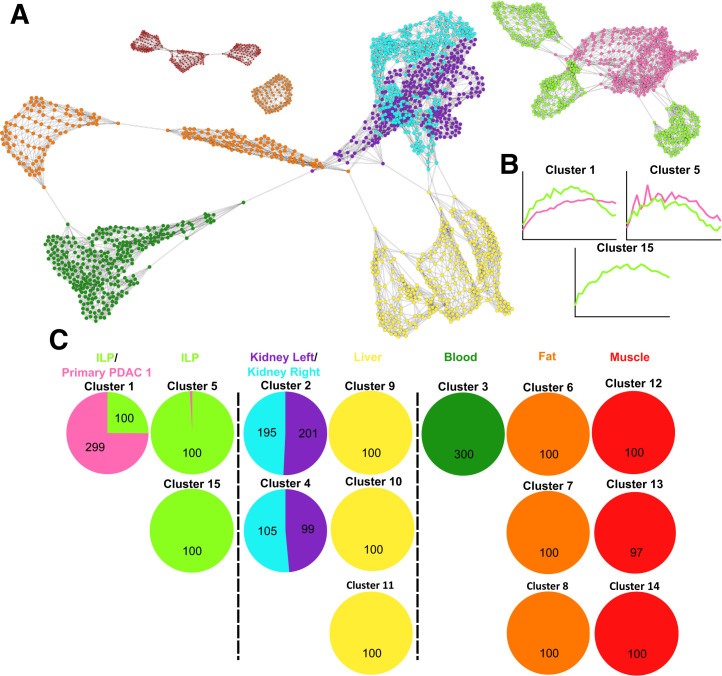
Individual PET scan–based network from case with PDAC and ILP. (A) Voxel network arrangement on which cluster analysis was performed. (B) Averaged time–activity curves from pathologic clusters containing voxels sampled from PDAC (rose) and ILP (green) regions. (C) Voxel cluster summary for all sampled regions. Network is based on Pearson correlation coefficient of >0.720, *k*–nearest neighbor of 9, Markov cluster granularity of 1.2, and minimum component size of 10.

**FIGURE 5.**
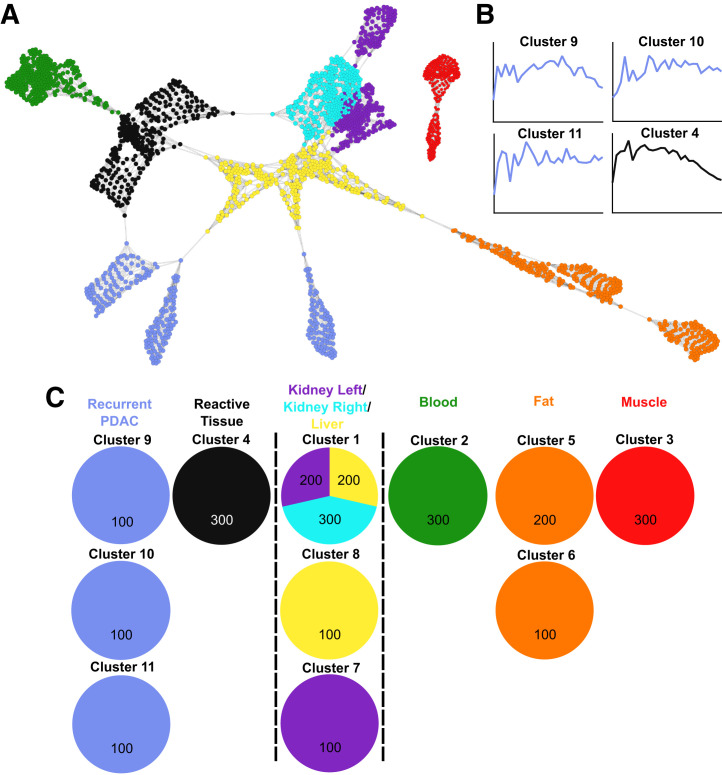
Individual PET scan–based network from case with RPDAC and PRT. (A) Voxel network arrangement on which cluster analysis was performed. (B) Averaged time–activity curves from pathologic clusters containing voxels sampled from RPDAC (blue) and PRT (black) regions. (C) Voxel cluster summary for all sampled regions. Network is based on Pearson correlation coefficient of >0.75, *k*–nearest neighbor of 8, Markov cluster granularity of 1.2, and minimum component size of 10.

In total, 47 individual networks were created. All individual networks are provided in Supplemental Appendix 1 (primary setting) and Supplemental Appendix 2 (recurrence setting). Figure 4 shows an example individual network and the identified clusters from a case with primary PDAC and ILP, and Figure 5 shows an example individual network and corresponding clusters from a case with RPDAC and PRT. Within all individual scan networks, voxels tended to arrange and cluster within the sampled VOI, apart from the left and right kidneys, which strongly coclustered (Figs. 4C and 5C). Networks typically arranged into healthy controls, elimination organs, and pathologic (malignant and nonmalignant) regions. Pathologies tended to cluster with high purity (>95% from the same VOI), although some examples had an overlap in malignant and nonmalignant pathologic voxels (4/12 patients with both malignant and nonmalignant pathologies; Fig. 4C; Supplemental Appendices 1 [patients 6, 9, and 25] and 2 [patient 39]). The average number of clusters per pathologic VOI was 1.6 (range, 1–3), indicating intralesional heterogeneity in most cases. Our analysis approach was able to largely differentiate between malignant and nonmalignant pathologies in the case of PDAC versus ILP (Fig. 4; Supplemental Appendix 1), as well as RPDAC versus PRT (Fig. 5; Supplemental Appendix 2). This differentiation was driven by slower clearance of FAPI within the malignant voxels (Figs. 4B and 5B).

### Combined Networks in Primary and Recurrence Settings

**FIGURE 6.**
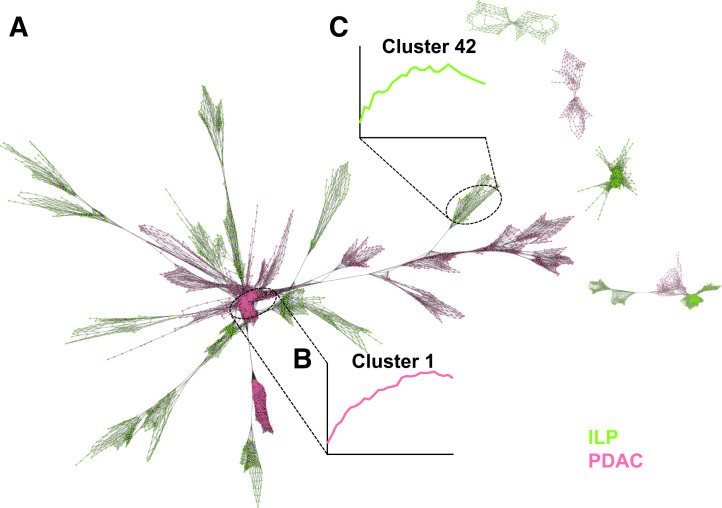
Combined PET scan network created from voxels sampled from all PDAC and ILP cases. (A) Voxel network. (B) Time–activity curve of central malignant core characterized by slow, minimal clearance. (C) Time–activity curve of nonmalignant pathology, exhibiting earlier and more pronounced clearance. Network is based on Pearson correlation coefficient of >0.950, *k*–nearest neighbor of 25, Markov cluster granularity of 1.3, and minimum component size of 100.

**FIGURE 7.**
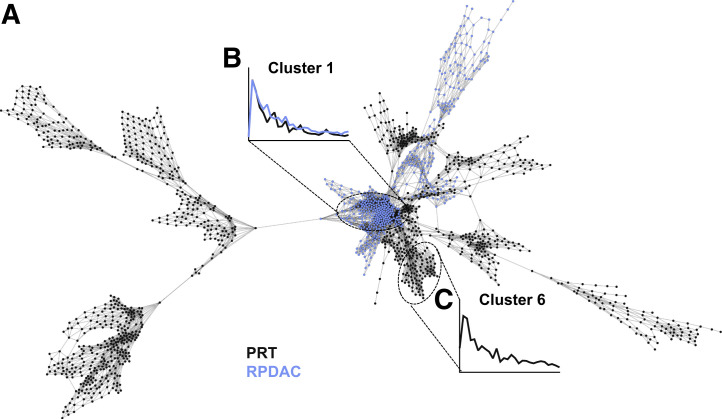
Combined PET scan network created from voxels sampled from all RPDAC and PRT cases. (A) Voxel network. (B and C) Time–activity curve of central malignant core (B) with marginally slower clearance than clearance of nonmalignant pathology (C). Cluster 1 is composed of 96% RPDAC voxels. Network is based on Pearson correlation coefficient of >0.950, *k*–nearest neighbor of 25, Markov cluster granularity of 1.3, and minimum component size of 100.

The combined network of the primary setting comprising 19 PDAC lesions of 16 patients (3/16 with 2 manifestations) and 21 ILP lesions of 19 patients (2/19 with 2 lesions) revealed a central core mainly consisting of PDAC clusters, which had a more sustained [^68^Ga]Ga-FAPI-46 signal toward the end of the scan relative to the ILP clusters, which tended to be peripheral to this malignant core (Fig. 6). A similar pattern was observed in the combined RPDAC (6 lesions) and PRT (9 lesions) network, although the difference in clearance rate is more marginal (Fig. 7). Across both combined networks, clusters almost exclusively clustered within their pathologic cluster, with minimal coclustering, such as the example shown in cluster 1 in Figure 7B (96% RPDAC).

## DISCUSSION

### Summary of the Results

Here, we present a fully data-driven approach for lesion clustering in a dataset of FAPI-avid primary and recurrent pancreas lesions, which were of unclear identity based on static FAPI PET imaging. In both primary and recurrence settings, benign and malignant pancreas lesions showed differential time–activity curves with delayed washout in PDACs compared with ILPs, similar to our previous findings on dynamic FAPI PET for malignant and benign pancreas lesions (16,17) and dignity assessment of pulmonary lesions (12). Fully data-driven, voxelwise clustering of single patients concordantly reflected these kinetic differences among FAPI-avid lesions with different identities by distributing voxels of benign and malignant pancreas lesions into distinct clusters with generally high purity. Moreover, clusters of pathologic pancreas lesions were clearly distinguishable from healthy control tissues and excretion organs, highlighting the biologic basis of the imaging, as well as clustering results. The strong coclustering between voxels sampled from the right and the left kidney highlights that if there is similarity in kinetic profiles, our approach can robustly detect it. Combined networks of pancreas lesions in both primary and recurrence settings showed visually easily discernable clustering of most malignancies and benign lesions, respectively, although a certain degree of visual overlap was observed for both settings. In summary, our results suggest that a digital biopsy approach followed by fully data-driven voxelwise clustering is a feasible, observer-independent approach to identify kinetic differences underlying the biology within healthy, benign, and malignant tissues at both individual and interindividual levels. This digital biopsy–based clustering approach offers a reproducible, observer-independent tool to assist in the assessment of pancreatic lesions.

### Network Analysis and Kinetic Modeling of Dynamic Data

Tracer uptake over time—mostly described by the time–activity curve of dynamic PET acquisition—can assist the differentiation of malignant and benign tracer-avid lesions, as demonstrated for tracers such as ^18^F-FDG (23,24), amino acids (25), prostate-specific membrane antigen (26), and recently FAPI (12,16). However, to date, the comprehensive, experience-requiring, and subjectivity-susceptible interpretation of the dynamic data prevents the extensive inclusion of dynamic PET data in routine clinical practice. An advantage of data-driven analysis over existing sophisticated evaluation methods for dynamic data, such as kinetic modeling, is that no prior assumptions are required. Avoidance of the need for aspects such as model selection and parameters removes the possible influence that these presumptions can have on the results. On the contrary, data-driven clustering may help to improve future kinetic modeling approaches by highlighting clinically relevant kinetic profiles, as well as facilitating the selection of relevant VOIs according to clustering results.

### Outlook: Potential Applications of Data-Driven Clustering in Nuclear Medicine

Beyond the differential diagnostic problems in pancreatic lesions, structured, data-driven clustering of sampled dynamic PET data appears to be a highly promising approach for the future of nuclear medicine. In general, this approach could help to address other unresolved diagnostic problems, such as differentiating between benign and cancerous lymph nodes in ^18^F-FDG–based lung cancer staging or prostate-specific membrane antigen–based prostate cancer staging, because distinct kinetic profiles have been described for these (27,28). If a large number of standardized samples were to be included, along with data-driven diagnostic information in terms of quantitative entity predictions, a tool similar to omics-based approaches already used in molecular pathology could become a reality (29). This approach appears particularly promising because the increasing availability of whole-body PET scanners and high-resolution dynamic PET data will enable the collection of large-scale dynamic datasets. In turn, these datasets could feed data-driven classifying systems like the 1 presented here and potentially pave the way toward automated diagnoses in nuclear medicine.

In the context of personalized medicine, intralesional subclusters may even enable more profound characterization of these lesions if clinical correlates of these subclusters, such as predictive and prognostic factors, can be identified. Furthermore, the degree of heterogeneity of the pathologic clusters may reveal insights into the heterogeneity of the pathologies themselves.

Although these initial results for image interpretation based on digital biopsy and voxelwise clustering in the context of FAPI PET for pancreatic lesions were promising, some limitations of this approach must be considered. One major limitation is the limited number of patients, particularly for the recurrence setting, included in the study. Thus, no definitive conclusions on the robustness or diagnostic value of this approach can be drawn based on the data presented here. However, on the basis of these initial findings, we consider that collection of a larger number of dynamic [^68^Ga]Ga-FAPI datasets for evaluation with this approach is highly warranted.

The acquisition of dynamic PET data over 60 min is time-consuming and not always easy to integrate into clinical routine. Although this article sets out an approach that can effectively differentiate between malignant and nonmalignant pathologic kinetic features, the potential to truncate the dynamic scan acquisition time or dual–time point imaging should also be explored in future studies to reduce the clinical demand placed on allocated scanner time caused by a 60-min dynamic scan. Another limitation is the retrospective, single-center nature of this analysis, which means a certain inhomogeneity among patients, especially in those with pretreatments, must be assumed. However, our retrospectively collected data may reflect the real-life experience of diagnostic uncertainty better than the highly selected, fully characterized patients of prospective trials.

Further limitations are the use of CT and clinical course instead of histologic confirmation as the reference standard for the pancreatic lesions of patients in the recurrence setting and the lack of evaluation of FAPI expression patterns in the pancreas. This lack of histologic confirmation could affect our interpretation of cluster purity in the recurrence setting, unlike in the primary setting, where 24 of 31 cases had histologic confirmation. Finally, only standard clinical PET parameters (SUV_max_ and SUV_mean_) of static imaging were analyzed here. Other PET parameters, such as the target-to-background ratio and the FAPI-positive volume of lesions, should be included in future projects on advanced imaging analysis of FAPI PET data.

## CONCLUSION

Static FAPI PET signal intensity, as well as the conventional, dynamic time-to-peak FAPI PET parameter, proved inconclusive for the entity assessment of pancreatic lesions in in primary and recurrence settings. In contrast, the present approach, which involves sampling of standardized digital biopsy followed by data-driven clustering analysis, demonstrated distinctive [^68^Ga]Ga-FAPI kinetics across pathologies. This enabled the differentiation and identification of healthy, nonmalignant pathologic, and malignant pathologic clusters. This sampling and analysis method is interpreter-independent and offers great potential for facilitating diagnosis in the future. As such, it warrants further investigation with larger datasets and across a wider range of pathologies to fully explore its diagnostic capabilities.

## DISCLOSURE

This work was funded by the German Federal Ministry of Education and Research (grant number 13N 13341) and by SULSA-Rheinland-Pfalz Research Collaboration Funding. Mark Macaskill is funded by the British Heart Foundation (RG/F/22/110093). Uwe Haberkorn has filed a patent application for quinoline-based fibroblast activation protein–targeting agents for imaging and therapy in nuclear medicine. He also has shares of a consultancy group for iTheranostics. No other potential conflict of interest relevant to this article was reported.

## Notes

KEY POINTS **QUESTION:** Can a digital biopsy approach with voxelwise network analysis and clustering of dynamic data improve FAPI PET-based assessment of unclear pancreatic lesions? **PERTINENT FINDINGS:** Although single static and dynamic FAPI PET imaging parameters could not sufficiently differentiate between malignant and benign pancreatic lesions, intra- and interindividual network analysis clusters showed high purity among malignant lesions, benign pathologies, and physiologic tissues. **IMPLICATIONS FOR PATIENT CARE:** Dynamic FAPI PET acquisition followed by digital biopsy and voxelwise network analysis with clustering can assist and facilitate the assessment of unclear, FAPI-positive pancreatic lesions.
